# The Effect of Comfort Nursing in Operating Room on Anxiety Depression and Postoperative Recovery in Thyroidectomy Patients With Coexisting Sleep Disorders

**DOI:** 10.62641/aep.v54i3.2238

**Published:** 2026-06-15

**Authors:** Wenjing Wu, Xiulan Huang, Qianwen Xu, Mei Dai, Jingjing Huang

**Affiliations:** ^1^Operating Room, Taizhou Second People’s Hospital, 225500 Taizhou, Jiangsu, China; ^2^Department of General Surgery, Taizhou Second People’s Hospital, 225500 Taizhou, Jiangsu, China

**Keywords:** comfort nursing in operating room, sleep disorders, thyroidectomy, anxiety, depression, quality of life

## Abstract

**Background:**

This study aimed to evaluate the effects of comfort nursing in the operating room on anxiety, depression and postoperative recovery in thyroidectomy patients with sleep disorders.

**Methods:**

This retrospective analysis included 156 propensity-score-matched patients undergoing thyroidectomy at Taizhou Second People’s Hospital (January 2023–September 2025). The patients were assigned to conventional care (n = 78) or comfort nursing (n = 78). Outcomes included Hospital Anxiety and Depression Scale (HADS), Pittsburgh Sleep Quality Index (PSQI), polysomnography (PSG), Quality of Recovery-15 (QoR-15), Visual Analogue Scale (VAS), thyroid cancer-specific quality of life (THYCA-QoL), recovery indices, complications and satisfaction.

**Results:**

Repeated-measures ANOVA revealed significant time, group and interaction effects for HADS-A, HADS-D and PSQI scores (all *p* < 0.001). Both groups improved postoperatively, with the comfort group showing greater reductions at 5 days and 4 weeks (*p* < 0.05). PSG parameters (total sleep time, sleep efficiency, REM%, sleep latency and microarousals) also improved in both groups, with superior outcomes in the comfort group (*p* < 0.05). Comfort nursing was associated with short times to first flatus and ambulation, reduced hospital stay, low choking incidence and high normal swallowing rate at 72 hours (*p* < 0.05). Complication rates did not differ between groups (*p* > 0.05). The comfort group achieved higher QoR-15 and lower VAS scores on days 1–3 (*p* < 0.001) and lower THYCA-QoL anxiety, psychological and sensory scores at 4 weeks (*p* < 0.001). Satisfaction was higher in the comfort group (84.62% vs. 69.23%, *p* < 0.05).

**Conclusions:**

Operating room comfort nursing alleviates anxiety and depression, improves sleep architecture, promotes early recovery and enhances quality of life and satisfaction in thyroidectomy patients with sleep disorders.

## Introduction

The detection rate and incidence trend of thyroid cancer have attracted widespread attention. Surgery, radioactive iodine and thyroid-stimulating hormone suppression remain the cornerstone of its treatment, amongst which surgery still occupies a central role [1]. However, owing to delicate neck anatomy, specific surgical positioning and patient concerns about surgical risks and unknown pathology results, remarkably perioperative anxiety and depression are common [2]. Perioperative adverse psychological states such as anxiety and depression may increase the risk of postoperative complications and reduce patient engagement during recovery, thereby affecting postoperative recovery [3]. Whilst conventional routine nursing can ensure perioperative safety, it still has certain limitations in terms of patient comfort experience, psychological support and individualised care. Comfort nursing in the operating room emphasises a patient-centred approach, strengthening communication, reassurance and individualised comfort support on the basis of routine verification, monitoring and safety management to alleviate the perioperative physical and psychological burden on patients and promote postoperative recovery [4].

Previous related studies primarily focused on patients undergoing general surgery or head and neck surgery, with relatively small sample sizes and outcomes mainly limited to subjective satisfaction and anxiety scores. For patients undergoing thyroidectomy with comorbid sleep disorders, objective sleep architecture parameters, particularly dynamic observations of polysomnographic measures, remain rarely reported. Moreover, existing study designs lack detailed definitions of intervention components [5, 6]. Additionally, patients with comorbid sleep disorders are prone to exacerbated anxiety, impaired sleep continuity and heightened stress responses during the perioperative period. Factors in the operating room setting, such as environmental stimulation, positional discomfort, information uncertainty and hypothermia, may further disturb neuroendocrine and sleep regulatory processes. Therefore, nursing interventions during the operative phase represent a critical window of opportunity.

In view of this situation, this study employed a retrospective cohort design to investigate the associations of comfort care in the operating room with anxiety, depression, subjective and objective sleep outcomes and postoperative recovery in patients undergoing thyroidectomy with comorbid sleep disorders. The aim was to provide data reference for optimising perioperative nursing care.

## Materials and Methods

### General Data

#### Inclusion and Exclusion Criteria

The inclusion criteria were as follows: (1) age ≥18 and ≤75 years; (2) postoperative pathological diagnosis of benign thyroid tumour, undergoing elective thyroidectomy under general anaesthesia; (3) diagnosis with insomnia disorder in accordance with the Diagnostic and Statistical Manual of Mental Disorders, Fifth Edition [7], with a Pittsburgh Sleep Quality Index (PSQI) score >7 [8]; and (4) complete and traceable medical records.

The exclusion criteria were as follows: (1) emergency surgery; (2) severe cognitive impairment; (3) severe cardiac, pulmonary, hepatic or renal insufficiency; (4) haematological diseases or uncontrolled severe endocrine disorders; (5) history of severe psychiatric disorders such as schizophrenia or bipolar disorder or long-term preoperative use of anxiolytic/antidepressant medications; (6) severe systemic infectious diseases; (7) comorbid with other diseases that may cause sleep disturbances, such as obstructive sleep apnoea syndrome, restless legs syndrome and narcolepsy; and (8) secondary insomnia resulting from pain, environmental changes or medication factors.

#### Patient Data

This retrospective cohort study initially included 210 patients with sleep disorders who underwent thyroidectomy at Taizhou Second People’s Hospital between January 2023 and September 2025. The classification of the two nursing models in this study was based on a phased update of the operating room nursing protocol at our hospital. Patients admitted between January 2023 and March 2024 received conventional operating room nursing and were defined as the conventional care group. Building upon the existing routine, our hospital fully implemented an enhanced protocol named the ‘operating room comfort care’ program between April 2024 and September 2025. Patients admitted during this period all received this new model of care and were defined as the comfort care group.

Core baseline variables including age, gender, operative duration, ASA classification and intraoperative blood loss were incorporated into the analysis model to control for potential confounders and enhance intergroup comparability. A 1:1 nearest neighbour propensity score matching with a calliper width of 0.02 standard deviations was applied. After matching, the standardised mean difference (SMD) for all core variables was below 0.1, indicating good balance in baseline characteristics. Finally, 156 successfully matched patients were included, with 78 in each group. This study strictly adhered to the ethical principles of the Declaration of Helsinki [9]. The research protocol was reviewed and approved by the Ethics Committee of Taizhou Second People’s Hospital (Ethics Review No.: ky2026-004-001), and informed consent was obtained from the patients.

### Methods

Conventional Care Group [10, 11, 12]:

(1) Preoperative Assessment and Education: Identity, procedure, side, skin markings and allergy history were verified before the patient entered the operating room. Past medical history, ASA classification, airway/bleeding risks, venous access and psychological status were assessed. Completeness of preoperative tests and imaging, NPO status and medication history were confirmed. Anaesthesia induction, neck positioning, monitor attachment and transfer processes were explained. Patients were informed about common postoperative discomforts such as dry/sore throat, voice changes, swallowing difficulty and incision pulling sensation. Removal of jewellery, dentures and contact lenses was managed. Patients were instructed on diaphragmatic breathing, relaxation techniques and pain communication. An actionable perioperative cooperation checklist was provided.

(2) Entry Verification and Aseptic Management: The surgical safety checklist was implemented at three timepoints: before anaesthesia, before incision and before leaving the operating room. Intravenous access was established, and baseline vital signs and temperature were recorded. Functionality of electrocautery, suction and monitoring equipment were checked. Electrode pads were placed away from bony prominences and damaged skin. Hand hygiene, skin preparation, draping, instrument/sponge/needle counts, specimen labelling and handover were performed. Timing and documentation of preoperative prophylactic antibiotics were verified. Assistance with positioning and securing tubes/airway during induction was offered. Eye lubrication/protection, dental protection and proper use of safety straps/restraints to minimise fall and traction risks were provided.

(3) Intraoperative Monitoring and Risk Control: Heart rate, blood pressure, oxygen saturation, respiratory parameters and temperature were continuously monitored. Assistance with fluid administration, analgesia/sedation and vasoactive drugs as ordered was provided. IV patency was maintained, infiltration was monitored and accurate intake/output recording was ensured. Shoulder rolls, head rings and positioning pads were used appropriately to prevent pressure injuries and peripheral nerve compression. Neck hyperextension, potential mucosal injury from intubation and incision bleeding were monitored. The surgeon was assisted in maintaining a clear operative field around the recurrent laryngeal nerve area. Final instrument/sponge/needle counts was performed post procedure, and consistency was recorded. Verbal handover of key points with the anaesthesiologist and surgeon was conducted. Lines and tubes were organised to prevent tangling or pressure.

(4) Postoperative Handover and Recovery Guidance: In the post-anaesthesia care unit (PACU), consciousness, airway patency, cough/swallow reflexes, neck incision drainage and bleeding were documented as per the handover checklist. Hoarseness, signs of laryngeal edema, dyspnoea, paraesthesia or tetany related to hypocalcaemia were assessed. Pain was evaluated using VAS and functional limitation, and analgesics and antiemetics were administered as ordered. Upon return to the ward, graded drinking and swallowing safety observation, early ambulation guidance, incision care and safe neck movement education were implemented. Patients were advised against forceful throat clearing and intense neck hyperextension. Follow-up appointments were clarified.

Comfort Care Group [13, 14, 15]:

(1) Environmental Comfort Management: Standard operating room temperature, humidity and ventilation were dynamically adjusted and maintained for a stable thermal environment. Operating table sheets and contact surfaces were ensured to be dry, smooth and warm. Warming blankets or forced-air warming devices were used as needed to reduce cold stress. Unnecessary door openings and staff movement were minimised to ensure stable air circulation and a calm environment.

(2) Communication for Stress Reduction and Emotional Support: Prior to entering the OR, the assigned nurse conducted thorough preoperative communication and psychological preparation, addressing the patient’s main concerns with explanations and encouragement. Patients were guided through relaxation techniques such as paced breathing and progressive muscle relaxation. Written instructions were provided if needed to enhance adherence. The patient was offered brief, consistent informational support upon entering the OR and guided through attention diversion during waiting periods. When possible, the patients were asked to familiarise themselves with the OR environment and transfer process beforehand to reduce anxiety from unfamiliar settings.

(3) Comfortable Positioning and Pressure Injury Prevention: The degree of neck extension was adjusted individually depending on cervical spine mobility and surgical field requirements. Pillow and shoulder roll height/thickness was individually adjusted to reduce posterior neck muscle tension. Gel pads or soft padding was used to protect pressure points (occiput, acromion, elbows, sacrum/coccyx and heels). Limbs were maintained in neutral alignment with secure but not restrictive fixation; arm abduction was kept within a safe range. A nonslip mat and antithrombosis elastic stockings (provided that contraindications have been ruled out) were used in combination to reduce the risks of positional slippage and lower limb venous stasis. Limb alignment, pressure points and changes in skin colour and temperature were rechecked at predetermined time points during surgery; if signs of compression were identified, then immediate communication with the surgeon was undertaken to allow adjustment.

(4) Temperature management and shivering prevention: Preoperatively, a warming blanket or forced‑air warming device was applied. Intraoperatively, continuous temperature monitoring was performed with trend recording to maintain target temperature management. Intravenous fluids, irrigation fluids and humidified oxygen were warmed in accordance with standard protocols to reduce hypothermia‑related shivering, vasoconstriction and stress elevation. Warming intensity was dynamically adjusted depending on temperature trends and anaesthesia duration. During postoperative transfer, coverage and warming continuity were maintained; at the time of handover in the PACU, temperature trends and warming measures were applied and recommendations for continued warming were explicitly communicated to establish a traceable temperature management record and implementation checklist. This protocol was developed by the operating room nursing management team, and all nursing staff received standardised training before implementation. The responsible nurse performed the interventions in accordance with the uniform procedure and documented them. The head nurse conducted weekly audits of nursing records and implementation to ensure the consistency and traceability of the intervention across patients in this group (Table 1).

**Table 1.** **Standardised protocol for comfort nursing in the operating room**.

Intervention domain	Specific measures	Implementation timing	Implementation standards
Environmental comfort management	Temperature control (21 °C–25 °C), humidity control (40%–60%), noise control (<45 dB), light adjustment	From 30 min preoperatively to end of surgery	Monitor and record every 30 min
Communication for stress reduction	Preoperative visit (15–20 min), intraoperative information support, immediate postoperative feedback	1 day preoperatively, before entering OR, during waiting period, during emergence	Standardised communication script
Positional comfort	Individualised neck extension angle, gel pad protection of pressure points and neutral limb positioning	From positioning to end of surgery	Assess skin colour/temperature every 30 min
Warming management	Preoperative warming blanket, continuous intraoperative temperature monitoring, fluid warming (37 °C)	From anaesthesia induction to PACU handover	Maintain core temperature ≥36 °C
Psychological support	Relaxation training guidance, tactile support (handholding), privacy protection	From before entering OR to end of surgery	Enhanced intervention for anxiety score ≥8

PACU, post-anaesthesia care unit; OR, operating room.

### Observation Indicators

#### Scoring Criteria

(1) Hospital Anxiety and Depression Scale (HADS) scores [16] were collected via the medical record system at baseline, 5 days postoperatively and 4 weeks postoperatively to assess anxiety and depression. The scale has two subscales (A for anxiety, D for depression) with 7 items each (score range 0–21 per subscale; scores 0–7: no anxiety/depression; 8–10: borderline; and 11-21: probable case). (2) Pittsburgh Sleep Quality Index (PSQI) [17] scores were collected via the medical record system. It includes 19 self-rated and 5 bed-partner-rated items across seven components. The total score ranges from 0 to 21, with high scores indicating poor sleep quality. (3) Postoperative Quality of Recovery-15 (QoR-15) scores [18] at postoperative days 1, 2 and 3 were collected via the medical record system. The QoR-15 comprises 15 items covering 5 dimensions. The score range for each individual item is 0–10 points, and the total score ranges from 0 to 150 points. The higher the score, the better the postoperative recovery. (4) Pain Visual Analog Scale (VAS) scores [19] on postoperative days 1, 2 and 3 were collected from the medical record system. The scores range from 0 to 10, with high scores indicating severe postoperative pain. (5) Thyroid disease-specific quality of life questionnaire (THYCA-QoL) scores [20, 21] at postoperative week 4 were collected from the medical record system. The questionnaire consists of 24 items, comprising seven multisymptom scales and six single-symptom items. Each item is rated on a four-point Likert scale, with raw scores ranging from 1 to 4. Domain scores were linearly transformed in accordance with the scoring manual of the European Organisation for Research and Treatment of Cancer, yielding a range of 0–100 for each domain. High scores indicate great symptom burden and poor quality of life.

#### Polysomnography (PSG)

PSG data were collected from the medical record system at preoperative baseline, postoperative day 5 (POD5) and postoperative week 4 (POW4). Recorded parameters included total sleep time, sleep latency, sleep efficiency, percentage of rapid eye movement (REM) sleep and number of arousals. Preoperative PSG was completed within 1 week before surgery. The POD5 assessment served as an early evaluation time point to reflect changes in sleep continuity and architecture during the acute recovery phase following thyroidectomy. During this period, common postoperative issues such as pain, throat discomfort and dysphagia are most prominent and have a relatively pronounced impact on sleep. The POW4 assessment was used to evaluate recovery status after the perioperative acute stress had largely subsided. For patients who remained hospitalised, standard PSG was performed in the in‑hospital sleep monitoring laboratory. For discharged patients, follow‑up PSG at POD5 and POW4 was scheduled in the outpatient sleep laboratory upon their return to the hospital. For patients with limited mobility or scheduling conflicts, a portable home sleep monitoring system, set up by trained technical staff under the support of the hospital’s sleep centre, was used to improve follow‑up compliance and temporal consistency. All examinations were performed and interpreted by the same sleep monitoring team following a standardised operating protocol.

#### General Information

Data were collected via medical record review. Two researchers independently extracted and verified information from the hospital’s electronic medical record, anaesthesia information and nursing information systems using a standardised form. The data included age, gender, body mass index (BMI), ASA classification [22], operative duration, intraoperative blood loss and education level. Postoperative outcomes were as follows: time to first flatus, time to first ambulation, length of hospital stay, incidence of drinking-associated choking within 24 hours, rate of normal swallowing at 72 hours, incidence of temporary recurrent laryngeal nerve injury, permanent recurrent laryngeal nerve paralysis, hypocalcaemia, inadvertent parathyroid removal and total complication rate. ASA Physical Status Classification: Patients with ASA grade I or II have good tolerance to anaesthesia and surgery, and anaesthesia typically proceeds uneventfully. Patients with ASA grade III are at a certain risk for anaesthesia; adequate preoperative preparation is required, and effective measures should be taken to actively prevent potential complications during anaesthesia. Patients with ASA grade IV are at an extremely high risk for anaesthesia; even with optimal preoperative preparation, perioperative mortality remains high. ASA grade V indicates a moribund patient who is not expected to survive without the operation; anaesthesia and surgery are both exceptionally hazardous, and elective surgery is not recommended. The patients included in this study were classified as ASA grades I–III.

#### Satisfaction

Patient satisfaction scores were collected from the medical record system using a scale developed by our institution. The satisfaction scale was developed by our hospital, and its content validity was evaluated by experts. The overall Cronbach’s alpha coefficient of the scale was 0.814. The scale consists of four dimensions, with a total score ranging from 0 to 100. Scores of 90–100 were defined as very satisfied, 75–89 as satisfied, 60–74 as neutral and scores below 60 as dissatisfied. The satisfaction rate was calculated as (number of very satisfied + number of satisfied) / total number of cases × 100%.

### Statistical Analysis

Data were processed by SPSS 26.0 (IBM Corp., Armonk, NY, USA). Continuous data conforming to normal distribution (assessed by Shapiro Wilk test) are presented as ($\bar{x}$ ± s). Repeated measures ANOVA was used for data collected at multiple time points. Independent samples t-test was used for pairwise comparisons between groups, and paired samples t-test was applied for intragroup pre–post comparisons. Data with a skewed distribution were described as median (interquartile range) M (P25–P75). Between-group differences were analysed by Mann–Whitney U test, and within-group dynamic changes were assessed using Wilcoxon signed-rank test. Categorical data were presented as n (%) and compared using Chi-square tests. A two-sided *p*-value < 0.05 was considered statistically significant. Exact *p*-values were presented to preserve information on the strength of evidence; values below 0.001 were reported as *p* < 0.001.

## Results

### Comparison of Baseline Characteristics

No significant differences in gender, age, BMI, ASA class, operative time, blood loss, education or surgical type were found between the two groups (*p* > 0.05, Table 2).

**Table 2.** **Comparison of baseline characteristics**.

Characteristic		Conventional care (n = 78)	Comfort care (n = 78)	t/χ^2^	*p*
Gender [n(%)]				0.471	0.493
	Male	23 (29.49)	27 (34.62)		
	Female	55 (70.51)	51 (65.38)		
Age (years)		48.96 ± 8.74	49.14 ± 8.32	−0.132	0.895
BMI (kg/m^2^)		23.86 ± 2.84	23.79 ± 2.91	0.152	0.879
ASA class [n (%)]				1.006	0.587
	Ⅰ	20 (25.64)	24 (30.77)		
	Ⅱ	50 (64.10)	49 (62.82)		
	Ⅲ	8 (10.26)	5 (6.41)		
Operative time (min)		95.63 ± 18.77	93.23 ± 20.23	0.768	0.444
Blood loss (mL)		42.52 ± 12.63	43.11 ± 15.85	−0.257	0.797
Education [n (%)]				1.187	0.553
	Primary or lower	6 (7.68)	9 (11.54)		
	Junior/Senior High	45 (57.69)	39 (50.00)		
	College or above	27 (34.62)	30 (38.46)		
Surgical type [n (%)]				0.277	0.871
	Total thyroidectomy	30 (38.46)	32 (41.03)		
	Near-total thyroidectomy	25 (32.05)	22 (28.21)		
	Thyroid lobectomy	23 (29.49)	24 (30.77)		

BMI, body mass index; ASA, American Society of Anesthesiologists.

### Comparison of HADS and PSQI Scores

Repeated measures ANOVA showed the statistically significant main effects of time and group and time-by-group interactions for HADS-A, HADS-D and PSQI scores (*p* < 0.001). Within-group comparisons confirmed significant decreases in these scores at postoperative day 5 and week 4 compared with those at baseline in both groups (*p* < 0.05). Between-group comparisons revealed that the comfort care group had significantly lower scores than the conventional care group at postoperative day 5 and week 4 (*p* < 0.05). The significant time-by-group interactions indicated that the trends in score changes over time differed significantly between the groups (*p* < 0.05, Table 3).

**Table 3.** **Comparison of HADS and PSQI scores ($\bar{x}$ ± s, points)**.

Group	HADS-A score			HADS-D score			PSQI score		
	Pre-op	Post-op 5d	Post-op 4wk	Pre-op	Post-op 5d	Post-op 4wk	Pre-op	Post-op 5d	Post-op 4wk
Conventional care (n = 78)	11.37 ± 0.86	9.05 ± 1.14*	8.85 ± 1.07*	10.25 ± 1.23	9.14 ± 0.98*	7.40 ± 0.85*	13.43 ± 1.52	11.10 ± 1.46*	9.15 ± 1.21*
Comfort care (n = 78)	11.41 ± 1.03	7.95 ± 0.85*^#^	6.41 ± 0.86*^#^	10.31 ± 1.11	8.35 ± 0.84*^#^	6.23 ± 0.69*^#^	13.38 ± 1.61	10.03 ± 1.35*^#^	6.45 ± 1.03*^#^
F	F_time_ = 364.771, F_group_ = 86.934, F_time×group_ = 121.863			F_time_ = 350.826, F_group_ = 101.224, F_time×group_ = 92.442			F_time_ = 312.412, F_group_ = 68.792, F_time×group_ = 54.120		
*p*	*p*_time_ < 0.001, *p*_group_ < 0.001, *p*_time×group_ < 0.001			*p*_time_ < 0.001, *p*_group_ < 0.001, *p*_time×group_ < 0.001			*p*_time_ < 0.001, *p*_group_ < 0.001, *p*_time×group_ < 0.001		

Note: HADS-A, Hospital Anxiety and Depression Scale-Anxiety subscale; HADS-D, Hospital Anxiety and Depression Scale-Depression subscale; PSQI, Pittsburgh Sleep Quality Index; Pre-op, preoperative baseline; Post-op 5d, postoperative day 5; Post-op 4wk, postoperative week 4. *p < 0.05 vs. Preop within group; ^#^*p* < 0.05 vs. Conventional Care group at same time point.

### Comparison of PSG Parameters

Repeated measures ANOVA indicated statistically significant main effects and interactions for all PSG parameters (*p* < 0.001). Within-group comparisons showed that at postoperative day 5 and week 4, both groups had increased total sleep time, sleep efficiency and REM sleep percentage and decreased sleep latency and microarousal frequency relative to their preoperative values (*p* < 0.05). Between-group comparisons demonstrated that the comfort care group had significantly better values for all PSG parameters than the conventional care group at both postoperative time points (*p* < 0.05 or < 0.001). The interaction effects confirmed significantly different trajectories of change between the groups (*p* < 0.001, Table 4).

**Table 4.** **Comparison of PSG parameters ($\bar{x}$ ± s)**.

Parameter	time	Conventional care (n = 78)	Comfort care (n = 78)	F	*p*
Total sleep time (min)	Pre-op	296.85 ± 24.41	293.04 ± 26.78	F_time_ = 355.201, F_group_ = 28.736, F_time×group_ = 15.893	*p*_time_ < 0.001, *p*_group_ < 0.001, *p*_time×group_ < 0.001
	Post-op 5d	354.52 ± 26.85*	378.74 ± 28.06*^#^		
	Post-op 4wk	382.02 ± 24.18*	419.63 ± 24.15*^#^		
Sleep latency (min)	Pre-op	74.41 ± 15.23	72.56 ± 16.91	F_time_ = 312.547, F_group_ = 22.941, F_time×group_ = 12.685	*p*_time_ < 0.001, *p*_group_ < 0.001, *p*_time×group_ < 0.001
	Post-op 5d	57.85 ± 12.02*	50.32 ± 9.66*^#^		
	Post-op 4wk	46.14 ± 8.74*	41.01 ± 6.98*^#^		
Sleep efficiency (%)	Pre-op	71.02 ± 3.85	70.89 ± 3.94	F_time_ = 410.329, F_group_ = 31.574, F_time×group_ = 18.295	*p*_time_ < 0.001, *p*_group_ < 0.001, *p*_time×group_ < 0.001
	Post-op 5d	75.85 ± 3.67*	79.97 ± 3.88*^#^		
	Post-op 4wk	83.14 ± 4.01*	85.15 ± 3.78*^#^		
REM sleep (%)	Pre-op	14.23 ± 2.11	14.18 ± 2.20	F_time_ = 105.842, F_group_ = 8.327, F_time×group_ = 4.672	*p*_time_ < 0.001, *p*_group_ = 0.005, *p*_time×group_ = 0.011
	Post-op 5d	16.58 ± 2.25*	17.74 ± 2.61*^#^		
	Post-op 4wk	17.84 ± 2.33*	18.86 ± 2.77*^#^		
Microarousals (n)	Pre-op	38.25 ± 4.85	39.11 ± 5.02	F_time_ = 485.173, F_group_ = 42.158, F_time×group_ = 25.417	*p*_time_ < 0.001, *p*_group_ < 0.001, *p*_time×group_ < 0.001
	Post-op 5d	29.63 ± 3.74*	24.12 ± 3.16*^#^		
	Post-op 4wk	21.15 ± 3.12*	17.15 ± 3.04*^#^		

Note: PSG, polysomnography; REM, rapid eye movement; Pre-op, preoperative baseline; Post-op 5d, postoperative day 5; Post-op 4wk, postoperative week 4. *p < 0.05 vs. Preop within group; ^#^*p* < 0.05 vs. Conventional Care group at same time point.

### Comparison of Postoperative Recovery Outcomes

The comfort care group had significantly shorter times to first flatus and first ambulation and hospital stay compared with the conventional care group. The incidence of drinking-associated choking within 24 hours was lower (*p* < 0.05) and the rate of normal swallowing at 72 hours was higher in the comfort care group (*p* < 0.01). The total incidence of complications (temporary/permanent recurrent laryngeal nerve injury, hypocalcaemia and inadvertent parathyroid removal) did not differ significantly between the groups (*p* > 0.05). The comfort care group exhibited significantly higher QoR-15 scores and lower pain VAS scores on postoperative days 1, 2 and 3 (*p* < 0.01 or < 0.001). At postoperative week 4, the comfort care group showed significantly lower scores on the anxiety, psychological and sensory subscales of the THYCA-QoL questionnaire (*p* < 0.001). No significant intergroup differences were found for the scores on the other subscales (*p* > 0.05, Table 5).

**Table 5.** **Comparison of postoperative recovery outcomes**.

Outcome measure		Conventional care (n = 78)	Comfort care (n = 78)	t/χ^2^	*p*
Time to first flatus (h)		18.91 ± 2.02	16.14 ± 1.98	8.649	<0.001
Time to first ambulation (h)		8.85 ± 1.10	6.79 ± 0.74	13.723	<0.001
Hospital stay (d)		5.85 ± 1.04	5.12 ± 0.78	4.959	<0.001
Choking within 24 h [n (%)]		15 (19.23)	6 (7.69)	4.457	0.036
Normal swallowing at 72 h [n(%)]		52 (66.67)	68 (87.18)	9.244	0.002
Complications [n (%)]					
	Temporary RLN injury	3 (3.85)	2 (2.56)		
	Permanent RLN palsy	1 (1.28)	0 (0.00)		
	Hypocalcaemia	2 (2.56)	1 (1.28)		
	Inadvertent parathyroid removal	1 (1.28)	1 (1.28)		
	Total	7 (8.97)	4 (5.13)	0.880	0.348
QoR-15 score (points)					
	Postop day 1	98.85 ± 10.36	108.41 ± 11.55	−5.442	<0.001
	Postop day 2	113.63 ± 13.74	122.05 ± 16.23	−3.497	0.001
	Postop day 3	123.47 ± 15.89	130.21 ± 14.74	−2.746	0.007
Pain VAS score (points)					
	Postop day 1	3.78 ± 1.05	3.44 ± 0.62	2.463	0.015
	Postop day 2	2.65 ± 0.84	2.03 ± 0.47	5.689	<0.001
	Postop day 3	1.99 ± 0.57	1.40 ± 0.28	8.205	<0.001
THYCA-QoL at 4wk (points)					
	Neuromuscular	43.85 ± 7.74	42.15 ± 6.83	1.454	0.148
	Voice	37.45 ± 5.52	36.94 ± 5.82	0.562	0.575
	Concentration	41.52 ± 4.96	40.36 ± 4.43	1.541	0.125
	Anxiety	46.85 ± 6.95	37.02 ± 6.22	9.308	<0.001
	Throat/Mouth	35.26 ± 4.51	34.63 ± 4.69	0.855	0.394
	Psychological	49.85 ± 6.85	34.12 ± 5.46	15.859	<0.001
	Sensory	36.63 ± 5.85	31.14 ± 4.74	6.440	<0.001
	Scar problems	28.98 ± 6.10	27.74 ± 5.85	1.296	0.197
	Feeling cold	32.56 ± 4.85	33.02 ± 4.63	−0.606	0.545
	Tingling hands/feet	32.63 ± 5.06	31.75 ± 4.85	1.109	0.269
	Weight gain	30.25 ± 4.62	29.82 ± 4.54	0.586	0.559
	Headaches	32.85 ± 4.51	32.05 ± 5.22	1.024	0.307
	Reduced sexual interest	34.51 ± 4.06	33.89 ± 4.52	0.901	0.369

Note: QoR-15, Quality of Recovery-15; VAS, Visual Analogue Scale; THYCA-QoL, Thyroid Cancer-Specific Quality of Life; RLN, recurrent laryngeal nerve; Postop, postoperative; h, hours; d, days; wk, weeks.

### Comparison of Satisfaction

The satisfaction rate was significantly higher in the comfort care group (84.62%) than in the conventional care group (69.23%) (*p* < 0.05, Table 6).

**Table 6.** **Comparison of satisfaction [n (%)]**.

Group	n	Very satisfied	Satisfied	Fair	Dissatisfied	Satisfaction rate
Conventional care	78	32 (41.03)	22 (28.21)	12 (15.38)	12 (15.38)	54 (69.23)
Comfort care	78	41 (52.56)	25 (32.05)	8 (10.26)	4 (5.13)	66 (84.62)
χ^2^						5.200
*p*						0.023

## Discussion

Thyroidectomy is a key treatment for thyroid disorders. The level of detail in perioperative care directly impacts clinical recovery and patient experience, making it a central focus of clinical nursing. In practice, patients often face challenges such as slow swallowing recovery and gastrointestinal dysfunction postthyroidectomy, which can prolong hospitalisation and increase burden. Comfort nursing has a certain positive effect on postoperative recovery and functional improvement in patients undergoing head and neck surgery [23]. This study showed that the comfort care group had significantly shorter times to first flatus and first ambulation and hospital stay, a lower incidence of choking within 24 hours and a higher rate of normal swallowing at 72 hours postoperatively compared with the conventional care group, suggesting that comfort nursing in the operating room may contribute to improved early recovery in thyroidectomy patients. This phenomenon may be related to the detailed aspects of comfort care, such as positional protection, environmental management, warming measures and postoperative recovery guidance, which can, to some extent, alleviate perioperative discomfort and promote early mobilisation and feeding recovery. No significant difference in the total incidence of complications such as recurrent laryngeal nerve injury and hypocalcaemia was found between the two groups, suggesting that the impact of comfort nursing in the operating room on these surgical-related complications remains unclear. Although the absolute number of complications was lower in the comfort care group, the low event rate precludes definitive conclusions at this stage, warranting further validation in large-sample studies.

Postoperative recovery quality is an important dimension for evaluating the effect of comfort nursing intervention in the operating room, and pain is a common complaint.

A systematic review by Holzer *et al*. [24] indicated that compassionate interventions help alleviate perioperative anxiety and depressive symptoms in patients. Wan *et al*. [25]found significantly lower postoperative VAS scores in caesarean section patients receiving comfort care. The present study showed that the comfort care group had significantly higher QoR-15 scores and lower pain VAS scores on postoperative days 1, 2 and 3, supporting the value of comfort nursing in enhancing postoperative experience. This phenomenon is mainly attributed to individualised positioning protection, pressure point cushioning and perioperative temperature management, which reduce intraoperative and early postoperative discomfort. In terms of emotional and psychological support, preoperative communication and explanation, continuous intraoperative attention and informational support may alleviate patients’ anxiety regarding the surgical procedure and prognosis, thereby improving their acceptance of the perioperative experience. Regarding recovery of independence, early postoperative ambulation guidance and rehabilitation education help patients promptly transition into an active recovery state. With respect to pain management, the combination of comfort nursing with postoperative discomfort assessment and recovery instruction may exert a positive effect on pain control and subjective recovery perception. Consistent with these findings, the present study showed that the VAS scores in the comfort nursing group were lower than those in the conventional nursing group on postoperative days 1, 2 and 3, suggesting that comfort nursing also has advantages in alleviating early postoperative pain. The VAS scores in both groups gradually decreased over time, which aligns with the general pattern of postoperative recovery; however, the comfort nursing group had lower scores at each time point, indicating that this nursing model may improve patients’ early postoperative pain experience. QoR-15 encompasses multiple dimensions, and its improvement reflects an optimisation of patients’ overall perioperative experience rather than being limited to any single component. Similarly, the reduction in pain scores may be associated with a combination of factors, including reduced discomfort, improved emotional status and enhanced compliance with recovery protocols.

In thyroidectomy patients with sleep disorders, perioperative sleep disturbance often manifests as prolonged sleep latency, reduced efficiency and increased microarousals. Disrupted sleep architecture can amplify pain perception and worsen negative emotions. PSG provides objective measures of sleep continuity and structure. Our results showed improvement in all PSG parameters at postoperative day 5 and week 4 in both groups, with greater improvement in the comfort care group. This finding suggests that comfort nursing in the operating room promotes sleep architecture restoration. The potential mechanisms include multidimensional stressor control: dynamic warming and noise/light reduction lowering sympathetic arousal and wakefulness drive; communication and privacy protection reducing uncertainty and hypervigilance; and postoperative pain management and early activity guidance improving nighttime comfort and reducing arousal risk. These factors synergise to improve sleep continuity and REM sleep, providing a physiological foundation for emotional stability and functional recovery.

Anxiety, depression and sleep disturbances often interact in patients with comorbid sleep disorders undergoing thyroidectomy. This study showed that at postoperative day 5 and postoperative week 4, the comfort nursing group had lower HADS‑A, HADS‑D and PSQI scores compared with the conventional nursing group, suggesting that comfort nursing improves negative emotions and subjective sleep quality. Furthermore, the observed reduction in HADS scores was approximately 2–3 points, which exceeds the previously reported minimal clinically important difference (MCID; approximately 1.7 points), indicating that this improvement may be clinically meaningful in addition to being statistically significant [26]. Dias *et al*. [27]also reported that patient-centred preoperative dialogue alleviates preoperative anxiety in patients undergoing major visceral surgery, and the present findings are consistent with this view. Although the time spent in the operating room is limited, this period represents a critical phase of concentrated perioperative stress exposure. Factors such as an unfamiliar environment, uncertainty regarding surgery and anaesthesia, cold exposure, noise disturbance and forced positioning may all increase patient vigilance and tension. Comfort nursing in the operating room—provided through targeted preoperative communication, intraoperative environmental optimisation, individualised positioning protection and continuous warming measures—may mitigate psychological stress and physical discomfort to some extent, thereby contributing to postoperative improvements in anxiety, depression and sleep. Pain, throat discomfort and dysphagia are common after thyroidectomy, and these issues affect postoperative sleep quality. Therefore, the beneficial effects of comfort nursing may also be indirectly mediated by alleviating early discomfort and improving sleep continuity. The present study did not measure biomarkers such as cortisol, inflammatory cytokines or autonomic nervous function; thus, the above mechanisms remain speculative and are based primarily on clinical observations and existing literature.

Thyroid disease-specific quality of life reflects the treatment’s lasting impact on daily living, with different dimensions responding variably to interventions. Ye and Zhao [28] observed improvement in multiple QoL dimensions using supportive psychological and continuous care in thyroid surgery patients. Wang *et al*. [29] emphasised the importance of psychological and sensory dimensions in patient-centred comfort management models. In our study, the comfort care group had lower scores on the anxiety, psychological and sensory subscales of the THYCA-QoL compared with the conventional care group at postoperative week 4, with no significant differences in other dimensions. This finding is consistent with the aforementioned research in terms of anxiety and psychological dimensions. Meanwhile, no between-group differences were observed in functional dimensions such as throat and voice. This selective improvement may be related to the intervention’s focus on emotional support and sensory discomfort control. In theory, intraoperative neck protection and nerve-sparing assistance should reduce voice issues. The lack of difference in voice scores may be due to the relatively limited surgical scope in this study, resulting in a ceiling effect for baseline voice risk. Throat/oral dimensions involve local inflammation and sensory changes. Although comfort care includes positioning to reduce mucosal pressure, direct surgical stimulation is unavoidable, and graded drinking benefited both groups. The significant psychological improvement is attributed to the three-phase synergy of preoperative communication reducing uncertainty, intraoperative environmental control minimising stressors and postoperative continuity providing support. Sensory improvement in numbness and tingling may be related to the padding reducing nerve pressure and early activity promoting circulation. The absence of differences in functional dimensions suggests that this nursing model has limited impact on structural functional recovery. In the future, nursing measures such as voice rehabilitation training can be incorporated to further optimise the nursing plan.

Nursing satisfaction is a comprehensive indicator of healthcare service experience, reflecting subjective approval of technical skill, communication and care. In this study, overall satisfaction was significantly higher in the comfort care group. Sun [30] found refined nursing improved satisfaction in orthopaedic surgery, and Zeng *et al*. [31] observed similar effects in colorectal cancer surgery. Quan [32] used environmental modification zoning, screens in ambulatory surgery to improve patient perception and satisfaction. A systematic review by Lundberg *et al*. [33] noted that waiting area improvements significantly reduced anxiety. Our results align with these studies, indicating that optimising the nursing experience positively impacts satisfaction. Reasons include integrating temperature, noise, lighting and privacy into standard workflow; enhancing the sense of being respected and receiving care; communication reducing perioperative uncertainty and increasing perceived professionalism; and fast recovery with less pain jointly improving the subjective perioperative experience.

Limitations. This study was a single‑centre investigation with a relatively concentrated sample source. Patient composition, perioperative management protocols and nursing team implementation patterns may have institutional specificity. Therefore, the external generalisability of the findings is limited. Although baseline comparisons between groups were performed to enhance comparability, this study could not fully control for selection bias and potential confounding factors as in a prospective randomised trial. Accordingly, the results must be interpreted as correlational evidence. Future multicentre prospective studies are needed to further validate the consistent efficacy and general applicability of comfort nursing in the operating room.

## Conclusions

Comfort nursing in the operating room can effectively alleviate perioperative anxiety and depression, improve subjective sleep quality and polysomnography parameters, promote early postoperative recovery and reduce pain levels in patients undergoing thyroidectomy with sleep disorders. It also demonstrated favourable advantages in promoting the anxiety, psychological and sensory-related quality of life dimensions whilst enhancing patient satisfaction with nursing care. The observed time-by-group interaction was statistically significant, indicating that the recovery trajectories differed between the comfort nursing and conventional nursing groups. Examination of outcomes at individual time points revealed that compared with the conventional nursing group, the comfort nursing group showed more pronounced improvement as early as postoperative day 5, and this advantage persisted through postoperative week 4. This finding suggested that the effect of comfort nursing is characterised by fast, early improvement and sustained benefit.

## Availability of Data and Materials

All experimental data included in this study can be obtained by contacting the corresponding author if needed.
